# Acoustic Inspection of Concrete Structures Using Active Weak Supervision and Visual Information

**DOI:** 10.3390/s20030629

**Published:** 2020-01-23

**Authors:** Jun Younes Louhi Kasahara, Atsushi Yamashita, Hajime Asama

**Affiliations:** Department of Precision Engineering, The University of Tokyo, Tokyo 113-8656, Japan

**Keywords:** acoustic inspection, defect detection, clustering, weak supervision, active query

## Abstract

Concrete structures are featured heavily in most modern societies. In recent years, the need to inspect those structures has been a growing concern and the automation of inspection methods is highly demanded. Acoustic methods such as the hammering test are one of the most popular non-destructive testing methods for this task. In this paper, an approach to defect detection in concrete structures with active weak supervision and visual information is proposed. Based on audio and position information, pairs of samples are actively queried to a user on their similarity. Those are used to transform the feature space into a favorable one, in a weakly supervised fashion, for clustering defect and non-defect samples, reinforced by position information. Experiments conducted in both laboratory conditions and in field conditions proved the effectiveness of the proposed method.

## 1. Introduction

Concrete structures are featured heavily in most modern societies. This is especially true for social infrastructures such as tunnels, bridges, and highways. Such large scale structures require regular inspection to prevent the propagation of damages caused by various factors such as rain and wind. The importance of such inspection work was underlined in recent catastrophic failures such as the collapse of the Sasago tunnel in Japan [1] or, more recently, the collapse of the Morandi bridge in Italy [2].

Inspection of large scale concrete structures is paramount to ensure the safety of their users and several methods exist for this task such as using capacitive transducers [3], microwave imaging [4], or embedded piezoceramic transducers [5].

**Figure 1:**
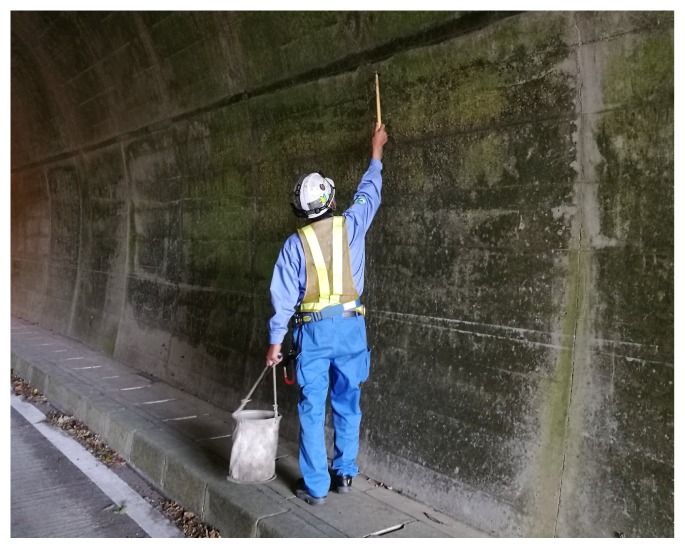
Hammering test conducted by a professional on the upper wall portion of a tunnel: only a simple hammer is needed, thus the popularity of this non-destructive testing method. However, there is the need of skilled operators to correctly differentiate hammering sounds and given the great population of structures in need of testing, automation is actively demanded. In addition, since it relies heavily on the operator’s skills, the final result remains subjective.

One popular method for the inspection of such structures is the hammering test. It consists of hitting the surface of the tested area with a hammer and using the returned impact sound to assess the presence of defects under the surface, as illustrated in Figure 1. This method is extremely popular due to its simplicity and non-destructive nature.

There are two factors complicating the inspection of concrete structures. Currently, inspection work is predominantly conducted by veteran human inspectors and there is a shortage of such skilled workers. On the other hand, the population of concrete structures reaching ages where inspection is critically required is ever increasing. Therefore, the automation of inspection methods such as the hammering test is highly demanded.

While there are some previous works employing direct methods [6], most of them used machine learning approaches. In [7], the sound of dragging chains across concrete surfaces was used with Linear Prediction Coefficients. In [8], Independent Component Analysis was used along a Radial Basis Function Neural Network. In [9], Ensemble Learning was used with time-frequency analysis. In [10], Partial Least Squares Regression was used to predict the gap in concrete-metal composite structures. While achieving high performance, such supervised learning approaches have an inherent dependency on the available training data. For concrete structures, such training data are neither available nor easy to produce.

The other end of the machine learning spectrum is called unsupervised learning. In [11], Mode Decomposition, Principal Component Analysis, and Independent Component Analysis were used for concrete bridge inspection. In [12,13], clustering methods were used. Those bypassed the issue of training data but involved strong priors in their design to compensate for the absence of training data, for which no mitigation would be possible.

Weakly supervised methods, also known as semi-supervised methods, are a mix of supervised and unsupervised methods. Such methods aim to achieve the best of both worlds. From the point of view of concrete structure inspection, such approaches are also extremely attractive since human involvement can be maintained during the actual inspection process. The work in [14] proposed an initial framework for weakly supervised inspection of concrete structures, later expanded by the addition of visual information in [15]. However, weak supervision was assumed to be provided by the human user on randomly chosen pairs of samples. However, this makes for weak supervision of inconsistent quality. Weak supervision quality greatly affects the performance of the resulting defect detection and, in most cases, it is possible to actively query the user for specific weak supervision.

Therefore, in the present paper, an active weakly supervised method reinforced by visual information for the inspection of concrete structures using acoustic methods is proposed.

## 2. Our Novelty and Overview of Proposed Method

The method of Louhi Kasahara et al. [13] was unsupervised, i.e., did not require supervision of any form by a human user, and used position information in order to reinforce the clustering in audio feature space. The proposed method is a weakly supervised method and therefore requires a human user to provide weak supervision on each considered dataset of hammering samples. The proposed method uses position information to both reinforce clustering in the feature space obtained by weak supervision and in the active query process, i.e., select pairs of samples to query the human user on.

The methods of Louhi Kasahara et al. [14] and Louhi Kasahara et al. [15] were weakly supervised. Louhi Kasahara et al. [14] only used weak supervision, as opposed to Louhi Kasahara et al. [15] and the proposed method that use position information to complement weak supervision. The method of Louhi Kasahara et al. [15] assumed that the human user would randomly select pairs of samples for weak supervision. This resulted in weak supervision of inconsistent quality and, thus, inconsistent defect detection performance. The proposed method actively queries the user: hammering samples are first processed and a selection process to decide which pairs of samples the human user should provide weak supervision on is proposed.

Our contributions can be summarized as follows:

- We explore an active weakly supervised method for the issue of defect detection in concrete structures.
- Previous work passively waited for a user to provide weak supervision and had no control over its quality. Our proposed method actively queries the user to increase the probability of obtaining quality weak supervision.
- Visual information is also employed to use the position of hammering samples in the active query as well as in the analysis in order to reinforce weak supervision results.

**Figure 2:**
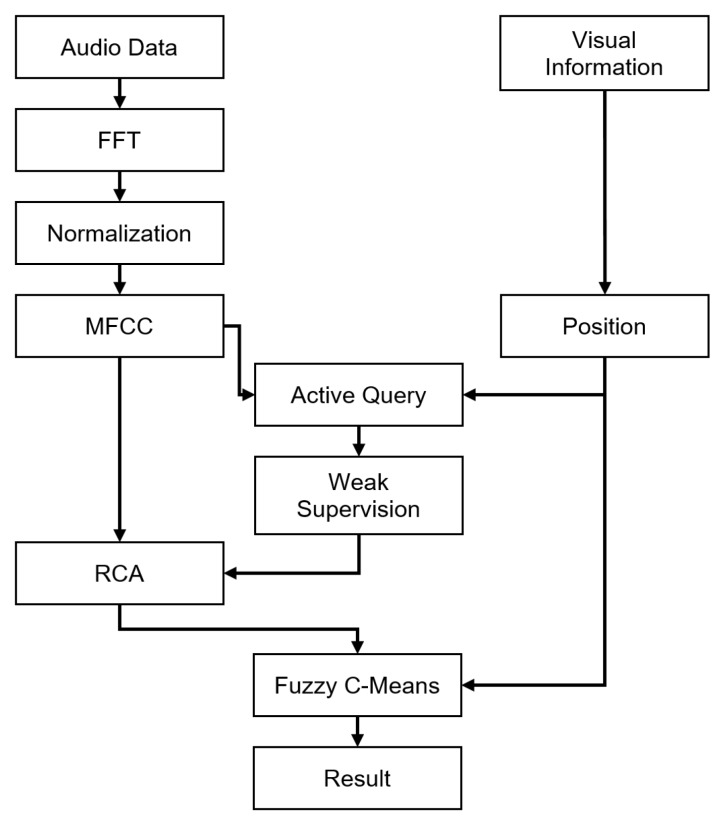
Overview of proposed method for active weakly supervised defect detection using hammering and visual information.

An overview of the proposed method is shown in Figure 2. During the hammering process, audio data are recorded and visual information is used to record the location of each hammering sample, i.e., the position on the tested structure where the hammer head contacted the concrete surface. Audio data are processed into Fourier spectrum using Fast Fourier Transform (FFT), normalized and finally converted into Mel-Frequency Cepstrum Coefficients (MFCC). Active query, based on both audio and visual information, is used to obtain quality weak supervision on MFCC samples and Relevant Component Analysis (RCA) [16] is used to learn an appropriate metric for discriminating defect hammering samples. Finally, Fuzzy C-Means, a fuzzy clustering algorithm, is used to discriminate defect and non-defect hammering samples.

## 3. Preprocessing

### 3.1. Fourier Spectrum and Position Information

Audio-samples are initially time-series data and not really suited for analysis. It has been long established in audio signal processing that frequency analysis, i.e., analysis using the Fourier spectrum, is a more suitable approach. The Fourier spectrum of an audio signal can be obtained using the Fourier transform.

The Fourier transform consists of decomposing a time-series function, i.e., a signal, into the different frequencies it contains. The Fourier transform of a signal in the time domain is a function in the complex domain, with its absolute value representing the amount of each frequency present in the original signal and its complex argument representing the phase offset. The absolute value of the Fourier transform, designated as simply *Fourier spectrum* in the remaining of this paper, is used as initial feature vector for a sound sample. Given a sound sample defined by $x=(x_{1},\ldots ,x_{d})$, its Fourier spectrum $a=(a_{1},\ldots ,a_{d})$ is defined as in Equation (1) using FFT, an algorithm computing the discrete Fourier transform.

(1) $$a_{j}=\left|\sum_{l=1}^{d}x_{l}e^{-\frac{2\pi i}{d}jl}\right|\;\;\;\;\;\;\;j=1,\ldots ,d$$

**Figure 3:**
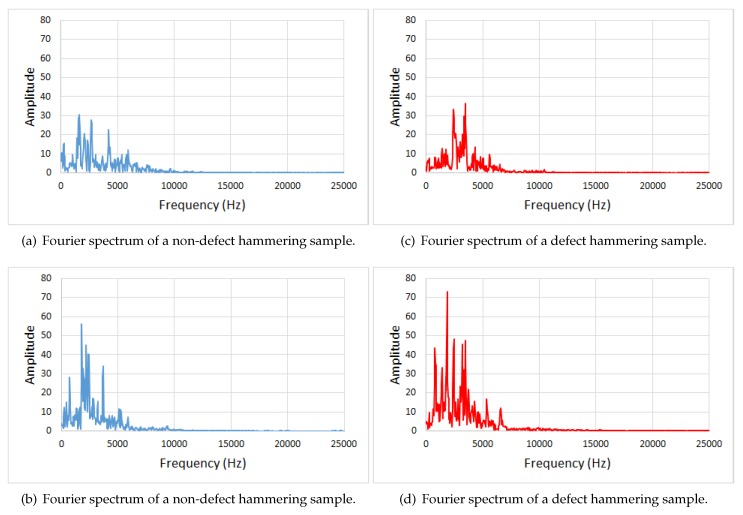
Fourier spectrums of hammering samples. It can be noted that features allowing discrimination of defect hammering samples are not obvious.

For illustration, Fourier spectrums of a couple of non-defect and defect hammering samples are shown in Figure 3.

### 3.2. Normalization

In the most general audio-based inspection setting, there is no assumption about the regularity of the input. In the case of the hammering test for concrete structures, there is no information about how much energy is used to hit the structure and to generate sounds. Especially in the case where a human conducts the striking motion, as opposed to a robot, there are no guarantees that the same amount of force is used for every audio sample and therefore correct comparison among those audio samples cannot be conducted.

To conduct comparison between audio samples, with $\bar{a}$ being the mean of the components of $a_{i}$ as defined in Equation (2) and *d* the dimensionality of a, a normalization to zero mean and unit variance is conducted as in Equation (3) to obtain the normalized Fourier spectrum $\tilde{a}=\left({\tilde{a}}_{1},\ldots ,{\tilde{a}}_{d}\right)$:

(2) $$\bar{a}=\frac{1}{d}\sum_{i=1}^{d}a_{i},$$

(3) $${\tilde{a}}_{i}=\frac{a_{i}-\bar{a}}{\sqrt{\frac{\sum_{i=1}^{d}{\left(a_{i}-\bar{a}\right)}^{2}}{d-2}}}.$$

### 3.3. Mel-Frequency Cepstrum Coefficients

The work presented in [12] showed the effectiveness of MFCC as feature vectors for hammering samples to the emulate the human audition’s ability to conduct the hammering test. MFCC is a hand-crafted feature vector originally built for speech recognition, devised with a strong understanding of how human beings perceive sounds. It is widely popular in the field of speech recognition as well as other related fields such as music information retrieval [17,18].

The main steps for building MFCC are as follows:

1. Calculate the periodogram estimate of the power spectrum.
2. Apply Mel filterbanks, i.e., ensemble of filters, to the power spectrum and sum the energy in each filter.
3. Calculate the logarithm of all filerbank energies.
4. Calculate the Discrete Cosine Transform (DCT) of the log filterbank energies.

The first step is to compute the periodogram estimate of the power spectrum as in Equation (4):

(4) $$P_{i}=\frac{1}{N_{window}}{\left|{\tilde{a}}_{i}\right|}^{2}.$$

Mel filterbanks are a set of $N_{filter}$ triangular filters, usually between 20 to 40, equally spaced in the Mel scale presented in Equation (5), where *f* is the frequency in Hertz. The Mel scale is an empirical scale tuned to the previously mentioned sensitivity of the human cochlea.

(5) $$M\left(f\right)=1125*\ln\left(1+\frac{f}{700}\right).$$

This filterbank is applied to the periodogram estimate of the power spectrum. This provides $N_{filter}$ energy values. Finally, the DCT of the logarithm of those energy values are calculated. The result is what are called MFCC.

For the sake of clarity, in the remainder of this paper, the MFCC of a hammering sample is denoted using simply x. Therefore, the dataset $D={\left\{X_{i}\right\}}_{i\in [1\ldots N]}$ is composed of hammering samples $X_{i}=\left\{x_{i},l_{i}\right\}$, with $l_{i}$ corresponding to the hit location on the tested structure.

## 4. Active Weakly Supervised Metric Learning

### 4.1. Active Query

The performance of weakly supervised methods is conditioned by the quality of the provided weak supervision. In the proposed method, RCA, a weakly supervised metric learning method, is employed. RCA learns an adapted metric for the clustering task at hand based on the weak supervision provided by a human user, i.e., hints on how the feature space should be. Concretely, weak supervision consists of what are called *constraints* or, more precisely, *must-links*, pairs of samples indicated as similar through querying the user. If those hints are distributed badly in the feature space, the transformation to the adapted feature space cannot be achieved with satisfying performance. In our previous work, it was assumed that the human user would randomly choose pairs of samples to provide as constraints. In the proposed method, the pairs of samples to query the user are actively selected in an effort to provide better performance.

The default metric, usually the Euclidian distance, is not satisfactory for discriminating defect hammering samples in the MFCC feature space. However, the shortcomings of a non-suited metric mostly appear over medium to short ranges, as local structures of the data are usually easier to discern than global ones. More concretely, if two samples are very similar to each other, their distance across various metrics would remain small and the difference between metrics would be relatively small. On the other hand, the correct metric should be able to capture accurately the distance between dissimilar samples. In terms of weak supervision, this means must-links between samples separated by a big default metric value are more likely to contribute to a meaningful change in the computed new metric.

Moreover, one particular aspect of hammering samples is that they are composed of audio and position data. This spatial correlation of hammering samples was shown to provide great help in defect detection performance [12]. Samples that are physically located near one another can be expected to usually have strong similarity since they resulted from hits of the hammer on similar concrete surfaces. The only case where this does not hold is for defect boundaries, where closely located samples do belong to different classes and are therefore dissimilar. This is not relevant for the scope of the proposed method since only similar pairs of samples are used as weak supervision. Therefore, it is desirable to obtain must-links for sample pairs that are located physically far from each another, i.e., weak supervision on distant pairs of samples for which the shortcomings of the default metric are most likely to be apparent.

To take into account the two concepts presented in the two previous paragraphs, we propose an active query scheme based on both distances in the audio space and physical space. Pairs of samples $\left\{X_{i},X_{j}\right\}$ are selected to be queried to a user based on the query selection probability P(i,j) defined as in Equation (6), where the first and second term of the numerator correspond to the default metric in MFCC and physical space, respectively, and the denominator is a normalizing term.

(6) $$P\left(i,j\right)=\frac{|x_{i}-x_{j}\left|+\right|l_{i}-l_{j}|}{\sum_{(k,l),k<l}|x_{k}-x_{l}\left|+\right|l_{k}-l_{l}|}.$$

The proposed active query scheme is described in Algorithm 1.
**Algorithm 1:** Pseudo-code of proposed active query scheme.
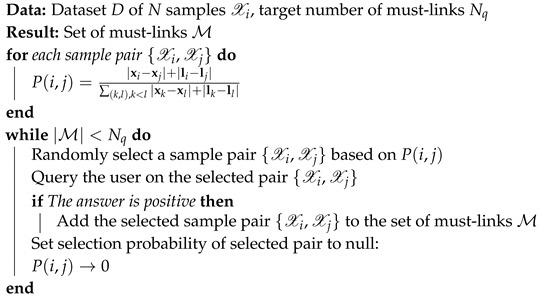


### 4.2. Relevant Component Analysis

RCA is a metric learning clustering method proposed initially in [16]. Several variants exists [19,20,21] and recently several successful applications have been reported [22,23].

Based on weak supervision, RCA computes a linear transformation of the feature, where the clustering task would be easier. Using must-links, a process akin to whitening is conducted. Given $N_{\mathrm{chunklet}}$ chunklets ${\left\{M_{l}\right\}}_{l\in [1\ldots N_{\mathrm{chunklet}}]}$, which are must-links regrouped using their transitive property, with ${\hat{m}}_{l}$ being the mean of elements in $M_{l}$, RCA can be divided into three steps:

1. For each chunklet, subtract its mean from each sample it contains.
2. Compute the covariance matrix $\hat{C}$ as in Equation (7), with $N_{\mathrm{total}}$ being the total number of elements contained in the chunklets.
  (7) $$\hat{C}=\frac{1}{N_{\mathrm{total}}}\sum_{j=1}^{N_{\mathrm{chunklet}}}\sum_{x_{i}\in M_{l}}\left(x_{i}-{\hat{m}}_{l}\right){\left(x_{i}-{\hat{m}}_{l}\right)}^{T}.$$
3. Compute the whitening transformation associated with this covariance matrix, i.e., the inverse square root of the covariance matrix, and apply it to the dataset as in Equation (8).
  (8) $$x_{new}={\hat{C}}^{-1/2}x.$$

## 5. Clustering with Position Information

Fuzzy C-Means is a suited clustering framework to incorporate spatial information along with the main data type [24] and has been successfully used with hammering data in [13]. It is a fuzzy clustering algorithm, meaning that samples belong to several clusters at the same time, with varying degrees expressed through *fuzzy membership coefficients*. Fuzzy C-Means in our proposed method is composed of two fuzzy membership coefficient update steps that are iterated until convergence has been reached. The algorithm is described in Algorithm 2.

### 5.1. Audio Feature Update Rule

The first update is the regular Fuzzy C-Means update, conducted on MFCC features only. For each sample $X_{i}$ toward each cluster center $c_{j}$, the corresponding fuzzy membership coefficient is noted $u_{ij}$. With ${\left\{c_{j}\right\}}_{\left[j=1\ldots K\right]}$, the cluster centroids, and *m*, a parameter controlling the fuzziness of the system, the update rule is conducted as in Equation (9):

(9) $$u_{ij}=\frac{1}{\sum_{l=1}^{N}{\frac{d(x_{l},c_{i})}{d(x_{j},c_{j})}}^{2/(m-1)}}.$$

### 5.2. Spatial Feature Update Rule

The compacity of defect and non-defect areas of the tested structure is expressed through the introduction of a spatial estimator, based on a spatial neighborhood $\mathrm{NB}(X_{i})$ for each sample $X_{i}$. This is defined using the position information of hammering samples, as in Equation (10). A spatial estimator $h_{ij}$ is then used to estimate the fuzzy membership coefficients of the considered sample based on its neighborhood, as in Equation (11), with $\left|\mathrm{NB}(X_{i})\right|$, the number of neighbors for sample $X_{i}$. This process enables keeping the same *hammering resolution* and offers a much more stable output compared to a simple smoothing step after clustering.

(10) $$\mathrm{NB}\left(X_{i}\right)=(X_{i}\in D\;\;|\;\;|l_{i}-l_{j}\left|\leq \gamma \right),$$

(11) $$h_{ij}=\frac{1}{\left|\mathrm{NB}(X_{i})\right|}\sum_{k\in \mathrm{NB}(X_{i})}u_{kj}.$$

Combination of the spatial estimator and regular Fuzzy C-Means update is conducted as in Equation (12), with *p* and *q* weighting exponents on each fuzzy components:

(12) $$u_{ij}\to \frac{u_{ij}^{p}h_{ij}^{q}}{\sum_{k}u_{kj}^{p}h_{kj}^{q}}.$$

The centroid update rule remains unchanged from the regular Fuzzy C-Means. Conversion to a crisp clustering is done by maximum membership. The whole process is summarized in Algorithm 2.
**Algorithm 2:** Pseudo algorithm of Fuzzy C-Means with Position Information.
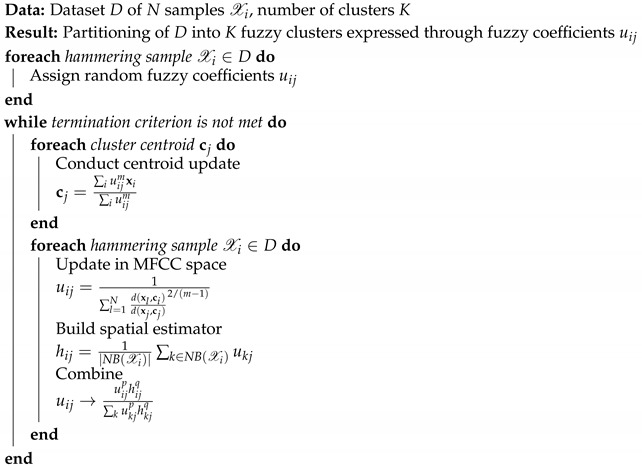


## 6. Experiments

### 6.1. Experimental Setup In Laboratory Conditions

**Figure 4:**
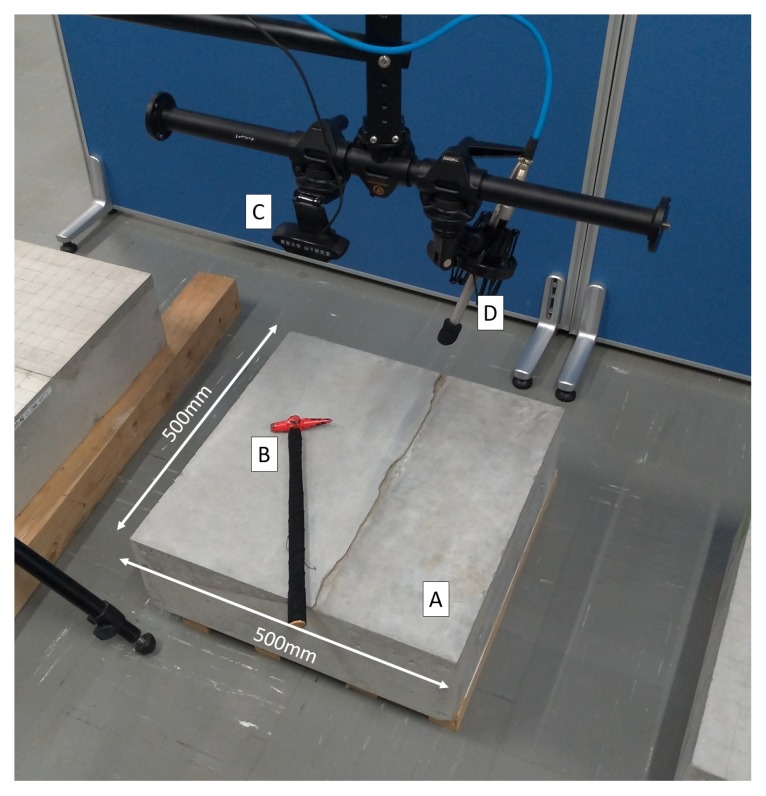
Experimental setup: concrete test block (**A**); hammer with red head for position recognition by image processing (**B**); web camera (**C**); and microphone (**D**).

The used setup is illustrated in Figure 4 and experiments were conducted on concrete test blocks containing various man-made defects to simulate natural ones. Standard concrete mixture ratios used in Japanese tunnels were also used for those concrete test blocks. Depending on the mixture ratios of concrete, the sound of the hammering test can vary greatly. For each type of structure, strict standards define the mixture ratio of concrete to be used and human inspectors are trained on each specific type of structure. Our proposed method does not rely on training data and, therefore, assuming that the human user providing the weak supervision has been trained on the corresponding concrete mixture ratio, it is applicable to all concrete mixture ratios.

Test blocks were hit at the upper surface on several locations, once per location. The used hammer was a KTC UDHT-2 (head diameter 16 mm, length 380 mm, weight 160 g), commonly used in hammering test by professionals and sound was recorded at 44.1 kHz using a Behringer ECM8000 microphone coupled with a Roland UA-25EX soundboard and a laptop for data analysis. Fourier Spectrums of length 1024 were obtained using FFT and MFCC were computed with 10 coefficients. The hammer head was painted in red and the location of each hammering sample was obtained by color-tracking the hammer head. In all experiments, *p* and *q* were set to the default value of 1, meaning equal contribution of audio data and visual information.

**Figure 5:**
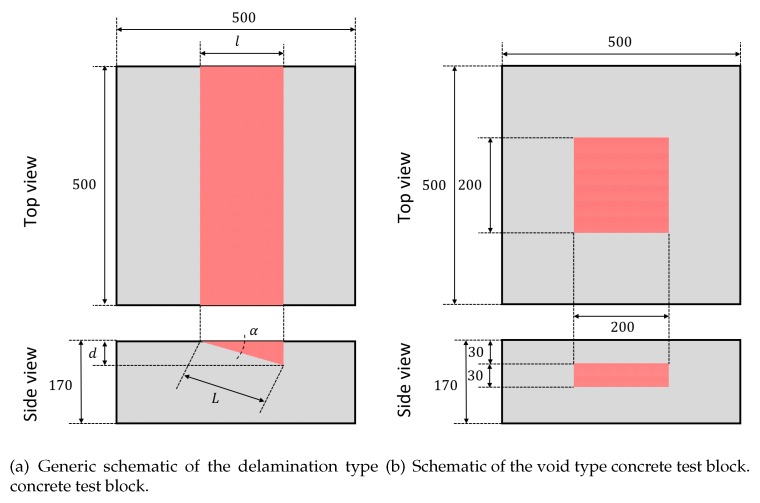
Schematic of the concrete test blocks containing man-made defects. Compared to natural defects found in the field, such concrete test blocks have the advantage of having the ground truth, i.e., location of defects, known in advance, without the need to destroy them, due to precise and elaborate fabrication processes.

Figure 5 shows a generic schematic of the blocks used in experiments. Red areas correspond to defect areas. Three scenarios were considered:

- Case 1 corresponds to a situation where the tested area of a concrete structure contains a single delamination. The dataset is composed of 462 samples: 272 non-defects and 190 defects. Referring back to Figure 5, α = 30 deg., *l* = 200 mm, *d* = 115.5 mm and *L* = 230.9 mm. The setting K=2 was used.
- Case 2 aims to simulate the presence of multiple delaminations in the tested area. Two concrete blocks containing each a single delamination were put together. This dataset is composed of 270 samples: 155 non-defects and 115 defects. Referring back to Figure 5, for the left side block α = 15 deg., *d* = 40 mm, *l* = 149.3 mm, and *L* = 154.5 mm. For the right side block α = 15 deg., *l* = 200 mm, *d* = 53.6 mm and *L* = 207.1 mm. The setting K=2 was used.
- In Case 3, one concrete block containing a delamination and another one containing a polystyrene cuboid to simulate a void were put together. This aims to simulate the co-existence of defects of different natures in a single tested area. This dataset is composed of 254 samples: 159 non-defects and 95 defects. The setting K=4 was used here since the two blocks were from different concrete batches.

### 6.2. Experimental Setup In Field Conditions

**Figure 6:**
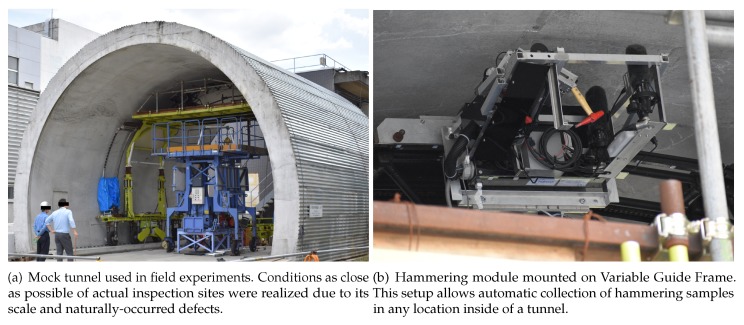
Field experimental setup using a mock tunnel and hammering module [13].

Field experiments in actual concrete structures currently in service present big logistic and legal issues. Therefore, experiments were conducted in a mock tunnel, as shown in Figure 6a. Its structure, fabrication process, and scale match the profile of small one way tunnels commonly found in Japan. In outdoor conditions, the mock tunnel differs from the previously presented concrete test blocks in the nature of its defects: they occurred naturally. Therefore, this mock tunnel can be considered to very accurately match actual inspection sites, while presenting the advantage of availability. Since the defects were not man-made here, their number and type were limited.

Two areas of interest were found in the mock tunnel, Area 1 and Area 2, both corresponding to a ceiling portion where a delamination was found. Furthermore, Area 1 presented extensive surface damage caused by rainwater leakage. Here, the ground truth of defect was obtained by the help of a veteran human professional, with expert knowledge of the hammering test. Great care was given during his inspection of the mock tunnel and, therefore, this human inspection result can be considered to be superior to what is usually conducted in actual inspection sites, where time is often a constraint. The setting K=2 was used for both Area 1 and Area 2.

Furthermore, the hammering was conducted in this mock tunnel using an automated hammering module, mounted on a system named *Variable Guide Frame*. An illustration is provided in Figure 6b [25,26,27]. This particular setup can collect hammering samples virtually anywhere inside a tunnel and the hammering motion was designed to mimic those of human inspectors. As reported in [25], the hammering module was designed to output consistent hammering at 0.2 J using a hammer head weighting 133.8 g, i.e., a hammering force of about 1.5 N.

## 7. Results and Discussions

**Figure 7:**
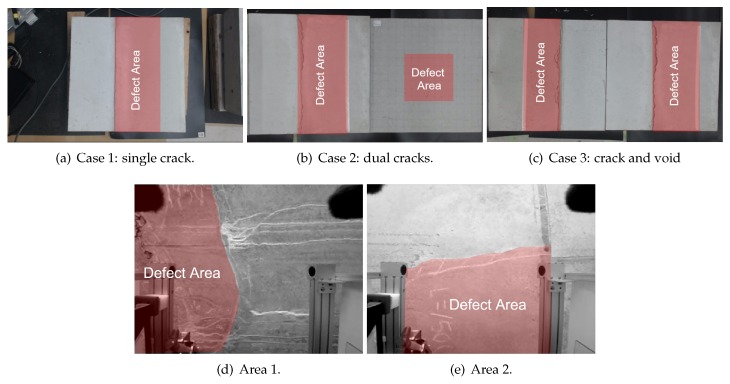
Picture of the considered cases in laboratory conditions and in field conditions. Red areas indicate defect areas.

**Figure 8:**
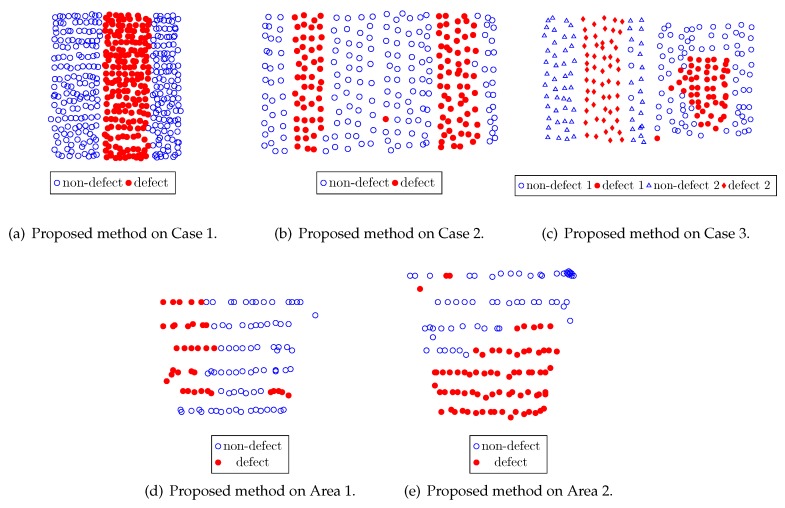
Example outputs of proposed method.

**Figure 9:**
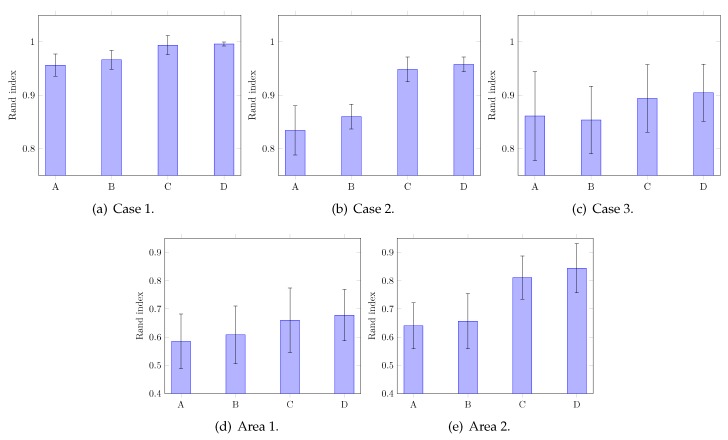
Performance evaluation of several weakly supervised methods for the hammering test in laboratory conditions: (A) method of Louhi Kasahara et al. [14]; (B) RCA with the proposed active query; (C) method of Louhi Kasahara et al. [15]; and (D) proposed method. The averages of 20 runs with sets of 20 must-links each are reported. Error bars corresponds to one standard deviation.

Figure 7 shows the tested concrete blocks and areas in the mock tunnel, with each defect area shown with a red overlay. Figure 8 illustrates the defect detection outputs using the proposed method. Figure 9 reports the performance obtained using the method of Louhi Kasahara et al. [14], RCA with the proposed active query method, the method of Louhi Kasahara et al. [15], and the proposed method.

In Figure 8a,b, it can be seen that the separation defect/non-defect hammering samples by our proposed method is excellent, only missing a couple of samples. In Case 3, shown in Figure 8c, the output of the proposed method shows an overspill of the void defect area. However, the two distinct defects, as well as the two distinct blocks, were rather well discerned. Regarding Area 1 and Area 2, the proposed method outputted the results reported in Figure 8d,e. The overall result is good. Misclassified samples are mainly located on the edge of the target area, hinting at possible variations induced by the hammering module. The defect/non-defect borders were however well discerned and the degraded surface condition of Area 1 did not seem to negatively affect the performance of our proposed method.

RCA with the proposed active query scheme should be put in direct comparison with Louhi Kasahara et al. [14], the only difference between those two methods being the active query scheme. For Cases 1 and 2, results in Figure 9a,b show that the proposed active query scheme achieves consistent, higher quality of weak supervision, resulting in a better performance in average and lower values of standard deviation.

The method of Louhi Kasahara et al. [15] employs both visual information and weak supervision, and it can be noted that this allows good performance in Cases 1 and 2, shown in Figure 9a,b. However, the results of Louhi Kasahara et al. [15] were lacking in consistency, shown by high values of standard deviation. The addition of the proposed active query scheme in our proposed method allows again to raise the average performance as well as increase the consistency of output.

Case 3, the results of which are reported in Figure 9c, seems to be a more difficult dataset. This is certainly due to the higher number of clusters since the two blocks were from different batches and defect types also vary. Without visual information, while the consistency of output was slightly increased by our proposed active query scheme compared to Louhi Kasahara et al. [14], the average performance slightly decreased. However, since the addition of visual information allows the proposed method to outperform the method of Louhi Kasahara et al. [15], it can be strongly suspected that the nature of errors was different when using the proposed active query scheme: those errors were easier to compensate for by the spatial estimator.

The proposed active query method allowed average performance increase for both Area 1 and Area 2, as shown in Figure 9d,e. Performance on the two datasets collected in field conditions are overall lower than in laboratory conditions. This is certainly due to the environmental noise in such outdoor settings coupled with the noise of the hammering module. The irregularity of the hammering grid pattern and the low number of samples compared to datasets obtained in laboratory conditions may have also have affected the performance, especially with regards to the spatial estimator.

## 8. Conclusions

An active weakly supervised method for defect detection in concrete structures using hammering, an acoustic inspection method, and visual information is proposed. By actively querying the human user, based on sample pair distance in audio feature space and physical space, the consistency of the quality of weak supervision was increased, resulting in an overall increase in performance. The proposed method can greatly help the human user provide adequate weak supervision and can be expected to be valuable for real inspections, where the number of samples to be considered can be colossal. Experiences in both laboratory conditions, using concrete test blocks, and field conditions, using a mock tunnel and automated hammering module, showed the effectiveness of the proposed method.

As future work, we would like to further explore the potential of active weak supervision. Some variants of RCA make use of dissimilar pairs [19] and an active query scheme for such pairs would certainly contribute to increase performance. We would also like to conduct additional experiments, at larger scale and with more varied defect configurations.
